# Natural Compounds as Promising Adjuvant Agents in The Treatment of Gliomas

**DOI:** 10.3390/ijms23063360

**Published:** 2022-03-20

**Authors:** Francesca Persano, Giuseppe Gigli, Stefano Leporatti

**Affiliations:** 1 Department of Mathematics and Physics, University of Salento, Via Per Arnesano, 73100 Lecce, Italy; giuseppe.gigli@unisalento.it; 2 CNR Nanotec-Istituto di Nanotecnologia, Via Monteroni, 73100 Lecce, Italy

**Keywords:** natural compounds, brain tumors, glioma tumors, glioblastoma, resveratrol, curcumin, epigallocatechin gallate

## Abstract

In humans, glioblastoma is the most prevalent primary malignant brain tumor. Usually, glioblastoma has specific characteristics, such as aggressive cell proliferation and rapid invasion of surrounding brain tissue, leading to a poor patient prognosis. The current therapy—which provides a multidisciplinary approach with surgery followed by radiotherapy and chemotherapy with temozolomide—is not very efficient since it faces clinical challenges such as tumor heterogeneity, invasiveness, and chemoresistance. In this respect, natural substances in the diet, integral components in the lifestyle medicine approach, can be seen as potential chemotherapeutics. There are several epidemiological studies that have shown the chemopreventive role of natural dietary compounds in cancer progression and development. These heterogeneous compounds can produce anti-glioblastoma effects through upregulation of apoptosis and autophagy; allowing the promotion of cell cycle arrest; interfering with tumor metabolism; and permitting proliferation, neuroinflammation, chemoresistance, angiogenesis, and metastasis inhibition. Although these beneficial effects are promising, the efficacy of natural compounds in glioblastoma is limited due to their bioavailability and blood–brain barrier permeability. Thereby, further clinical trials are necessary to confirm the in vitro and in vivo anticancer properties of natural compounds. In this article, we overview the role of several natural substances in the treatment of glioblastoma by considering the challenges to be overcome and future prospects.

## 1. Introduction

Noncommunicable diseases are known as long-lasting, slow-progressing diseases. These are the leading cause of adult mortality and morbidity worldwide. Of the noncommunicable diseases, cancer is one of the most terrifying diseases, a public health problem, and it is the second leading cause of death after cardiovascular disease in developed countries and the third leading cause of death in developing countries [1]. In this context, brain cancer is one of the most important cancers as its incidence is increasing worldwide and has attracted considerable attention in recent decades due to its low survival [2]. There are several studies that indicate a trend towards an increase in the incidence of this cancer [3]. The cause of central nervous system (CNS) malignancies is generally unknown. In terms of morbidity and mortality, malignant brain cancer is a catastrophic disease in adults and is the second leading cause of death in children [4]. Primary malignant brain tumors, although they represent only 2% of tumors, are related to severe disability and a high risk of death. The average survival time for each age is 9 months and the 5-year survival rate is less than 25% [5]. Some patients who survived more than 5 years developed chronic disabilities [6]. Brain tumors are heterogeneous and, in 2016—considering molecular abnormalities as diagnostic criteria—the World Health Organization (WHO) classified brain tumors into diffuse astrocytoma, other astrocytic tumors, oligodendroglial tumors, ependymal tumors, and other gliomas [7].

### 1.1. Glioma

Gliomas comprise 80% of malignant brain tumors and approximately 30% of all CNS tumors. Glioma is an extremely heterogeneous group of cancers that arise from the supporting cells of the brain [8]. More than 18,500 newly diagnosed cases and 13,000 deaths due to malignant glioma are reported annually in the United States [9]. The WHO classifies glioma into astrocytoma, ependymoma, pilocytic astrocytoma, and oligodendroglioma [7]. The histopathological characteristics—in particular the levels of nuclear polymorphism, the increased mitotic activity and cellularity, as well as the existence of neovascularization and necrosis—define a further system of classification of the tumor grade (I–IV) [10]. Additionally, current advances in next-generation sequencing have made it possible to identify genetic abnormalities in glioma that have been used to add another layer to glioma diagnosis [11]. Yet a recent WHO reclassification also considers molecular characteristics including mutations in the genes encoding isocitrate dehydrogenase 1 and 2 (IDH1/2) and TP53, in addition to the hypermethylation of the MGMT promoter and the loss of heterozygosity of chromosomes 1p and 19q [12]. The stage of glioma has a major impact on the patient’s prognosis [13]. Ependymoma and pilocytic astrocytoma (grade 1) typically represent slow-growing benign tumors; nevertheless, when they become malignant, a patient’s prognosis is poor [14]. For patients with grade II astrocytoma, life expectation is limited to 5–15 years [15]. Grade III astrocytoma often develops until grade IV astrocytoma, which is secondary glioblastoma, and has a 5-year survival rate of 30% [16]. Oligodendroglioma has a better outcome with a 5-year survival rate of 80% [17]. Whereas grade IV glioma (glioblastoma) is the most aggressive form of glioma, is characterized by a 5-year survival rate of 5%, and accounts for approximately 15% of all primary brain and CNS cancers and 55% of all gliomas [18].

### 1.2. Glioblastoma

Glioblastoma is a CNS (brain or spinal cord) malignant cancer resulting from astrocytes. It is one of the most common primary brain cancers, having incidence rates of 2.05 per 100,000 patients in the UK and 3.19 cases per 100,000 patients in the US [19]. To date, glioblastoma therapy includes surgical resection of the tumor, radiotherapy, and adjuvant chemotherapy with temozolomide [20]. However, complete surgical resection of gliomas is extremely difficult, due to local invasion and infiltration of the surrounding tissue; consequently, the tumor recurs causing the death of patients with glioblastoma [21]. In addition, the existence of the blood–brain barrier (BBB) further reduces the effectiveness of chemotherapy by hindering the delivery of chemotherapeutic agents to the CNS. In addition, the efficacy of chemotherapy drugs may be further reduced by the onset of (multi-) drug resistance [22]. Finally, although rare, extracranial metastases can extremely complicate the treatment [23]. Current therapy only minimally improves the median survival time of patients, from 12 months to 14.6 months, while the 5-year survival rate of treated patients is less than 10% [24]. As a result, there is an imperative need for the development of new therapies that are targeted and effectively effective in the treatment of glioblastoma [25]. Typically, the challenges raised by glioblastoma are found in the molecular and genetic signaling pathways happening in this type of cancer [26]. Genetic mutations in glioblastoma presents genes deletion, encoding cyclin-dependent kinase inhibitors (such as CDK2NA), amplification of epidermal growth factor receptor (EGFR) and cyclin-dependent kinase genes (such as CDK4), and the silencing of the O-6-methylguanine-DNA methyltransferase (MGMT) gene [27]. Such genetic alterations happen in cell cycle progression, upregulation of cellular mechanisms conducting to the promotion of proliferation (e.g., via Akt/mTOR signaling), self-perpetuating hyperinflammation, chemoresistance, tumor metastases, angiogenesis, and metabolic variations (known as the Warburg effect) [28]. At the same time, the effectors of apoptosis and autophagy are largely downregulated or inhibited. Therefore, conventional anticancer therapies aim above all to reverse this imbalance between death and growth, through the upregulation of apoptosis and the inhibition of proliferation [29]. The extreme molecular complexity and the challenges posed by chronic diseases, which include brain tumors, have prompted some doctors to adopt a holistic approach [30]. Due to the poor results of current treatment strategies, it is critical to improve treatment outcome [31]. Lifestyle medicine is dependent upon lifestyle factors (such as physical activity, the environment, and diet) together with general health maintenance aiming to reduce the risk factors related to chronic diseases [32]. An essential component of lifestyle medicine is natural food compounds. Through daily diet or over-the-counter supplements these compounds can be taken [33]. The use of dietary nutrients to improve prognosis for patients with glioma is a poorly investigated but highly attractive alternative due to their allegedly favorable safety profiles and reduced costs. While in vitro studies are promising, they have yet to be tangibly replicated in clinical trials [34]. Therefore, this review aims to assess the current state of understanding the role of some natural compounds in glioma as a foundation from which to build on a future application of these compounds in the prevention and treatment of glioma.

## 2. Natural Compounds in the Therapy of Glioma

Conventional anticancer therapies, including chemotherapy and radiotherapy, perform their cytotoxic action, essentially causing damage to the DNA of cancer cells [35]. However, there are several limitations when these treatments are applied as a single therapy due to the high heterogeneity that characterizes solid tumors and the deregulation of the different cell signaling pathways [36]. Glioblastoma, due to the heterogeneity of the tumor, its aggressive invasiveness into surrounding tissues, and the presence of BBB, is extremely difficult to treat [37]. An effective strategy for the treatment of glioblastoma can be represented by multimodal therapeutic approaches, which involve the combination of different therapies or therapeutic agents with different molecular mechanisms, in order to exert a better cytotoxic effect on tumor cells [38]. The multimodal therapeutic approach, in particular, can act effectively by making the DNA of tumor cells sensitive, using a first agent, to the harmful effects of a second agent [39]. Recently, there have been several epidemiological studies that have evaluated the role of various natural compounds in influencing the development, progression, and metastasis of cancer [40]. For many years, the use of products derived from nature has been of great interest to man, in the preservation of the state of health, for the improvement of physical and mental health, and in the prevention of diseases [41]. Several studies have reported the radio/chemoprotective role of some natural compounds in combination with radiotherapy and chemotherapy and/or synergistic effects in alleviating complications associated with radiotherapy/chemotherapy and increasing the efficacy of cancer therapies [42]. In addition, some of them can cross the BBB and this property is one of the main considerations for the development of therapies for the CNS [43]. The antioxidant nature and chemopreventive potential against cancer has been recognized for a wide range of natural compounds, including curcumin, epigallocatechin (EGCG), and resveratrol (RES) [44]. The main focus of our review work is to discuss the effect of these natural compounds in the prevention and treatment of glioblastoma.

### 2.1. Curcuminoids

Curcumin and its derivatives are plants secondary (poly) phenolic metabolites forming the curcuminoid family. Chemically speaking, curcuminoids are diarylheptanoids, characterized by a chain consisting of seven carbon atoms that connects two substituted aromatic rings [45]. These polyphenols can decrease proliferation and promote apoptosis, mitochondrial dysfunction, and cell cycle arrest in nervous system tumors. In addition, they can significantly modulate inflammation and angiogenesis of the tumor [46]. Furthermore, curcumin—which is derived from the South Asian turmeric plant—has shown inhibitory effects on glioblastoma [47].

#### 2.1.1. Curcumin

**Figure 1:**
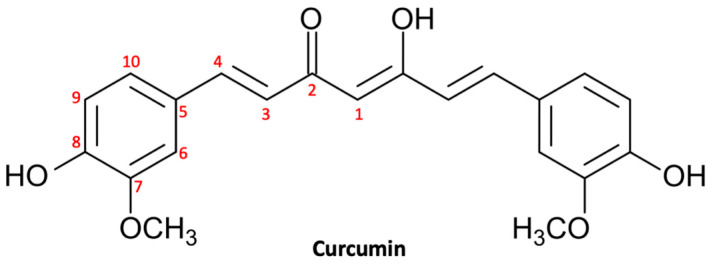
Molecular structure of curcumin.

Curcumin, also known as turmeric, is a natural polyphenol (Figure 1) extracted from the rhizome of *Curcuma longa* and was introduced as a culinary spice in Europe in the 14th century [48]. Curcumin is a natural compound that has been used in traditional Chinese and Indian Ayurvedic medicine for centuries to treat infections, allergies, respiratory disorders, liver disorders, and rheumatism [49]. Currently, curcumin is largely used for its multiplex biological activities, which include anti-inflammatory activity, antioxidant activity, stem cell modulation, regulation of mitochondrial homeostasis, and neurogenesis [50]. Consequently, curcumin could be useful in the treatment of a large range of diseases, including metabolic, inflammatory, and cardiovascular ones [51]. In recent decades, natural compounds—and curcumin in particular—have gained growing interest in both experimental and preclinical studies for their beneficial effects on the central nervous system (CNS) [52]. In fact, curcumin has shown multiple neuroprotective effects thanks to its anti-inflammatory, antioxidant, and anti-protein aggregation properties [53]. In addition, there are growing studies that support the chemopreventive, antiproliferative, antiinvasive, and antiangiogenic potential of curcumin, suggesting a potential utility in the treatment of neoplasms, including glioma [54].

##### Curcumin Pharmacokinetics

The component with the greatest biological activity of turmeric is curcumin, and it comprises about 2–8% of the volume of the preparation. Systemic bioavailability is secondary to first pass metabolism [55]. Intestinal metabolism is also implicated in the poor bioavailability of curcumin through sulfation and glucuronidation. Doses of approximately 3.6 g were detected in the colorectal tissue [56]. However, this would appear to be a local effect as transport through the intestinal mucosa is extremely reduced and the molecules entering the bloodstream are subjected to rapid chemical changes in the blood, liver, and other organs [57]. A single orally administered dose of 500–8000 mg of curcumin could not be detected in human serum 1 to 4 h post administration. The in vitro and in vivo stability of curcumin is extremely poor, with a half-life of approximately 5 min. Excretion happens through the biliary tract and, to a much lesser extent, via the renal system [58]. In view of the reduced bioavailability of curcumin at a systemic level, it is hypothesized that the plasma concentration of curcumin after oral administration is so low compared to LC50, curcumin may not exert a tumoricidal action outside of gastrointestinal tumors. Furthermore, there is limited evidence to suggest that curcumin is able to cross the BBB in humans; nevertheless, its exact mechanism is not clearly described [57].

##### Antitumor Properties of Curcumin in Glioma

**Figure 2:**
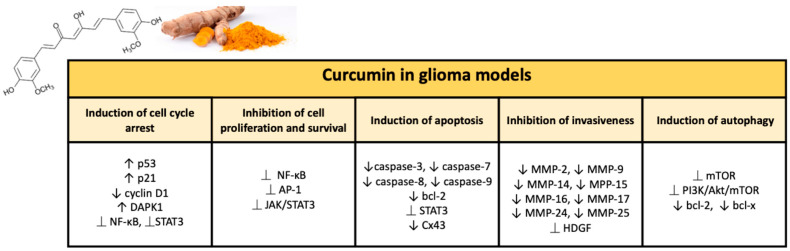
Major effects of curcumin on glioma models in preclinical studies.

Numerous studies have demonstrated the chemotherapy and radiation sensitizing potential of curcumin in various tumors, including glioma. Curcumin is able to act through multiple mechanisms thanks to modulation of the activity of transcription factors and regulating the expression of genes implicated in malignant transformation and cell survival [59]. Curcumin’s ability to target various pathways is linked to its complex and unique chemical structure. The present natural polyphenol, also called as diferuloylmethane (1,7-bis(4-hydroxy-3-methoxyphenyl)-1,6-hepadiene-3,5-dione; C_21_H_20_O_6_), is characterized by three reactive functional groups, such as two groups phenolics and 1,3-diketone [60]. These two phenolic groups are found to be essential for the multiple biological activities of curcumin, which make this natural substance a flexible and attractive therapeutic option [61]. Here, we briefly provide an overview of the broad range of neuroprotective activities of curcumin in glioma (Figure 2).

##### Inhibition of Cell Proliferation and Survival

NF-κB is a transcription factor, responsible for regulating the expression of various target genes implicated in the main biological and cellular processes, such as proliferation, inflammation, and the survival immune response [62]. NF-κB is constitutively overexpressed in glioblastoma and its aberrant activation is connected to EGFR deregulation (which promotes tumor cell proliferation and survival) and the PI3K/Akt/mTOR signaling pathway [63]. Equally, the excessive constitutive activation of c-Jun N-terminal kinase (JNK)/transcription factor AP-1 is related with the infiltration, resistance to therapies, and growth of glioblastoma [64]. AP-1 is a gene expression upstream modulator of MMP, thus regulating the invasiveness of glioblastoma [65]. Studies that have been conducted in vitro using human (U87MG, U373, T67, and T98G) and rat (C6) gliobastoma cell lines revealed that curcuminoids (i.e., demethoxycurcumin (DMC)) and curcumin result in reduced cell survival by suppression of NF-κB and inhibition of the AP-1 signaling pathway. This does not allow the constitutive activation of Akt and JNK [66,67,68]. The inhibition of the AP-1 signaling pathway is dependent on curcumin, at the same time, is causes a suppression of the transcription of MMPs, repressing the invasive character of glioblastoma cells [69]. In addition, curcumin-induced inhibition of NF-κB signaling enhances the antitumor activity of various therapeutic agents (including the microtubule stabilizing agent paclitaxel (PTX) and the alkylating agent nimustine hydrochloride (ACNU)) against glioblastoma [67,70]. A recent report has stated that curcumin is able to hinder the aggressiveness of glioblastoma in vitro through the inhibition of the JAK/STAT3 pathway [71]. Curcumin is capable of significantly suppressing glioblastoma cell proliferation via modulation of the JAK/STAT3 pathway in both primary and recurrent human glioblastoma cell lines [72]. Specifically, the antiproliferative activity was attributed, at least in part, with a decrease in intracellular STAT3 levels, with consequent reduction in the transcription of the c-Myc gene (cell cycle regulator) and of the Ki-67 marker (proliferation marker) [72,73]. In addition to the anti-proliferative action, the inhibition of the JAK/STAT3 signaling pathway induced by curcumin is strongly correlated with a significant inhibition of cell migration and therefore of invasiveness of the glioblastoma [74]. At the same time, TMZ and DMC synergistically inactivate JAK/STAT3 signaling in human glioma cells, and this explains the significant suppression of cell proliferation and the apoptosis induction in a combined treatment [75].

##### Induction of Cell Cycle Arrest

Several studies show that curcumin is able to exert anti-proliferative effects on glioma cells through the modulation of RB1/CDK4/p16INK4A and TP53/MDM2/MDM4/p14ARF signaling. These are two main cell cycle regulation pathways, which are frequently found altered in glioblastoma [76]. Curcumin significantly blocks cell growth and proliferation by suppressing cell cycle progression in different human glioma cell lines. In this regard, Liu et al. have reported that curcumin induces cell cycle arrest in G2/M in a p53-dependent manner. Indeed, p53 protein levels in U251 glioma cells treated with curcumin were augmented, upon induction of p21 (cell cycle regulator)/CDK inhibitor and tumor suppressor ING4, giving rise to cell cycle blockade [77]. Curcumin treatment, likewise, increases the expression of p53 and p21 and suppresses the RB and cdc2 pathways in DBRTG glioma cells [78]. Furthermore, another work conducted on U87MG cells has shown that curcumin determines the promotion of cell cycle arrest in glioblastoma cells by downregulation of cyclin D1, together with upregulation of p21 [79]. Surprisingly, however, this happens independently of p53 function, as it is based on the activation of Egr-1 (transcription factor) via the ERK and JNK/MAPK/Elk signaling pathways [80]. On the other hand, the curcumin-induced transcription of p21 fails in human cell line U87MG transfected with Egr-1 siRNA [81]. A recent work, with the aim of tracing the molecular mechanisms underlying curcumin-mediated cell growth suppression, reported that U251 cells treated with curcumin were blocked in the G2/M phase of the cell cycle following the increase tumor suppressor death-associated protein kinase 1 (DAPK1) expression. Furthermore, it is interesting to note that this effect is accompanied by inhibition of the STAT3 and NF-κB pathways, together with activation of caspase 3. Indeed, siRNA-mediated knock-down of DAPK1 determines an attenuation of the inhibition of NF-κB and STAT3 induced by curcumin, thereby preventing caspase 3 mediated apoptosis [82].

##### Induction of Autophagy

Curcumin determines a significant activation of autophagy (ATG), for this reason the potential beneficial action of this substance have been evaluated in different conditions of ATG deficiency. An example is represented by glioblastoma, which, due to a significant upregulation of mTOR, presents occlusion of the autophagic pathway [83]. There have been several studies that have demonstrated the ability of curcumin to exert its anticancer effects through inhibition of mTOR signaling, which among other effects determines the activation of the ATG pathway [84]. Through the modulation of various upstream regulators of the mTOR pathway—including the phosphatase and tensin (PTEN)/Akt homologue, the neuron the precursor cell expressed developmental downregulated protein 4 (NEDD4), the protein kinase activated by adenosine monophosphate (AMPK) and IκB β kinase (IKKβ)—curcumin induces ATG [85,86]. Furthermore, regardless of the molecules upstream of mTOR, another mechanism proposed for the activation of ATG induced by curcumin is the interruption of the mTOR-raptor interaction [87]. There are several in vitro works that have shown that curcumin is able to effectively prevent cell proliferation in glioblastoma by disturbing the mTOR pathway. Aoki et al., in this respect, reported that curcumin develops G2/M blockade of the cell cycle by promoting mTOR-dependent ATG in two neoplastic glioma cell lines (U87-MG and U373-MG). This was confirmed by an enhancement in LC3 immunoblotting, an increased red fluorescence signal by using flow cytometry upon acridine orange (AO) staining and the ultrastructure of the ATG vacuoles. Oppositely, suppression of the anticancer effects of curcumin was shown upon administration of the ATG inhibitor 3-methyladenine (3-MA) or recombinant human full-length active Akt1 protein (rAkt1) [88]. Maiti et al. demonstrated similar results in both rats (F98) and mice (GL261). In these species, the ATG vacuoles and ATG markers levels number of glial tumor cells (such as, Atg5, Atg7, Beclin-1, LC3A/B, and p62) were increased upon administration of curcumin. It is important to note that the levels of total p-PI3Kp85, PI3Kp85, Akt, p-Akt, p-mTOR, and mTOR were reduced following administration of curcumin. All of this confirms that this natural polyphenol is able to significantly activate the pathway of ATG dependent on mTOR [89]. This confirmed what was shown by Zanotto-Filho et al., who reported that curcumin could block constitutive activation of the PI3K/Akt/mTOR pathway, giving rise to a considerable viability reduction in in vitro glioblastoma cells. However, curcumin does not modify the phenotype of healthy astrocytes, thus suggesting that this natural compound selectively targets glioma cells [90,91]. Moreover, an increase in ATG levels developed by curcumin happens in conjunction with downregulation of cell survival markers such as the mitochondrial cellular protection protein bcl-x and bcl-2. This is most probably caused by the cytostatic/anti-proliferative effect of curcumin on glioblastoma cells [91,92].

Curcumin, through the induction of ATG, also eliminates the tumorigenicity of GSCs (glioblastoma cancer stem cells), a subpopulation of cancer cells that present stem-like characteristics, pluripotency, greater self-renewal, and clonogenic potential [93]. Specifically, GSCs are responsible for the onset of glioblastoma, progression, tumor regrowth after surgical resection, and therefore for the patient’s relapse. GSCs are also resistant to standard treatments and contribute to poor prognosis for patients with glioblastoma [94]. Consequently, therapeutic approaches aimed at eradicating these cells represent promising strategies against glioblastoma. Among the different pathways which support the metabolism and oncogenic properties of GSCs, mTOR-dependent ATG has a crucial role [95]. While finely tuned mTOR signaling is essential for the normal development of the CNS, GSCs instead exploit the improper activity of mTOR to fuel tumor growth and infiltration [96]. Indeed, these cells show significant upregulation of mTOR, resulting in marked suppression of ATG. There is clear evidence that curcuminoids and curcumin are able to counteract the malignant phenotype of glioblastoma and aggression by targeting ATG within GSCs [97]. The administration of curcumin, in addition to the improvement of ATG in GSC, creates multiple effects. For example, curcumin is involved in invasiveness of GSCs by downregulation of Akt/mTOR activity and in the suppression of stem-like tumorigenic characteristics [98]. This is relevant, since the ATG defect contributes to desensitization to the normal differentiation signals of GSCs and GSCs caused the enhanced therapeutic resistance and invasive potential [59]. In this regard, Zhuang et al. showed that curcumin causes the suppression of stem-like characteristics of GSCs by stimulating ATG-dependent differentiation of GSCs either in vitro either in vivo. In particular, the upregulation of neural markers (such as, Tuj1, Olig2, and βIII-tubulin) and the marked decrease in clonogenic capacity of GSC and self-renewal happen together the induction of ATG upon curcumin administration. This ATG-dependent pro-differentiating effect was also evidenced in vivo in nude mice carrying intracranial glioblastoma xenografts. The tumor sections extracted from mice treated with curcumin, and analyzed by transmission electron microscope (TEM), showed a significant augmentation in the number of autophagosomes and LC3 immunofluorescent spots. All these effects revealed a noticeable burden and increased mouse survival. As reported by the in vitro data, the double effect of curcumin on the stemness and differentiation of GSCs in xenograft tumors was reversed by treatment with 3-MA [99]. One of the principal factors inducing glioblastoma neoplasia is the strongly invasive potential of GSCs. GSCs, unlike non-stem cancer cells, easily infiltrate and migrate in the surrounding healthy brain parenchyma [100]. Hyperactivation of the Akt/mTOR pathway is known to support the highly invasive phenotype of GSC, while inhibition of mTOR results in a reduction in both mRNA, protein levels, and matrix metalloproteinase (MMP) activity MMP-9 and MMP-2, which enhance tumor invasiveness by degradation of the extracellular matrix [96]. As proof of this concept, the induction of mTOR-dependent ATG following the administration of curcumin significantly compromises the migration of GSCs in vitro, as well as their ability to invade the brain parenchyma in vivo [101]. Taken together, these data confirm that the effect of curcumin on GSC is strictly related to ATG induction, further reinforcing the idea that ATG-dependent differentiation, invasion inhibition, and migration arrest induced from curcumin may show promise in adjunct therapy of glioblastoma.

##### Promotion of Apoptosis

The antiproliferative effects of curcumin are partially caused by the activity of pro-apoptotic pathways. Several studies show that curcumin-induced G2/M cell cycle arrest often happens together with promotion of caspase-mediated cell death [102]. In human glioblastoma cells, curcumin causes apoptosis via a caspase-dependent pathway. Actually, treatment with curcumin induces an increase in the expression of pro-apoptotic proteins—such as caspase-7, caspase-8, caspase-3, and caspase-9—which start and induce an apoptotic process cascade [103]. Although the molecular mechanisms have yet to be clarified, curcumin has been reported to trigger different pro-apoptotic events and also an increase in caspase activity [104]. In fact, curcumin induces a cleavage of the nuclear enzyme of the poly (ADP-ribose) polymerase (PARP-1) and the fragmentation of DNA in human CHME glioma cells. Moreover, loss of mitochondrial membrane potential and increased ROS production are reported in curcumin-treated cells compared to controls [105], suggesting the induction of the apoptotic cascade. At the same time, curcumin induces an anti-glioblastoma effect by suppression of anti-apoptotic signals, as confirmed by the augmented Bax:Bcl2 ratio in different human glioblastoma cell lines [103]. Survivin is a protein belonging to the apoptosis inhibitor (IAP) family of proteins; it is expressed in embryonic tissues, and is upregulated in human tumor cells, while it is not present in most normal tissues [106]. Survivin performs multiple functions—including the inhibition of apoptosis and the regulation of the cell cycle, as well as having demonstrated to induce chromosomal instability [107]. In a recent work, 144 patients with glioblastoma underwent whole genome sequencing and the prognosis of the group with high survivin expression was poor, compared to those with lower expression [108]. Patient-derived glioblastoma cells and established human glioblastoma cell lines (U51, U87, U235) treated with 25 μM curcumin have demonstrated an increased expression of p38, JNK (via c-Jun) and phosphorylated ERK at 1 and 6 hours. The phosphorylated ERK inhibits STAT3 by dephosphorylation at Tyr705 and phosphorylation at Ser727, with the inactivation of STAT3 and the inability to translocate to the nucleus with following reduced expression of IAP2 and surviving [109]. Human glioblastoma U373 cells treated with increasing concentrations of curcumin in DMSO and studied by flow cytometry showed selective induction of apoptosis for lower concentrations (10 μg/mL) and similar levels of apoptosis and necrosis for higher concentrations (20 μg/mL) [110]. Carbobenzoxy-Leu-Leu-leucinal (MG132) is a proteasome inhibitor involved in the degradation of connexin 43 (Cx43), a gap junction protein associated with increased glioma invasiveness. Overexpression of Cx43 was assessed in TMZ-resistant cells. Exposure of TMZ-resistant glioblastoma cells to 10 μM of curcumin resulted in a two-fold (4% to 8%) increase in apoptosis, resulting in a 40% reduction in Cx43 expression; furthermore, subsequent exposure to MG132 inhibited the degradation of Cx43, involving the ubiquitin-proteasome pathway in the degradation of Cx43 [111]. Furthermore, curcumin has been shown to enhance the cytotoxic and apoptotic-promoting action of TMZ and etoposide, with the downregulation of mRNAs encoding p53 and the upregulation of BAX-Bcl2 in U87MG cells and T98G [112].

##### Inhibition of Invasiveness

Matrix metalloproteinases (MMPs) have been involved in tumor invasiveness. MMPs are zinc-dependent proteolytic endopeptidases that permit tumor cells to become locally invasive through degradation of extracellular matrix proteins, cell adhesion molecules, and growth factor binding protein [113]. MMPs have been reported to allow for disseminated tumor growth. Furthermore, MMP-2 should be required for vasculogenesis and angiogenesis by expression of vascular endothelial growth factor (VEGF) A and coexpression of VEGF receptor 2 (VEGFR2) [114]. It has been shown that MMP-9 is required for vasculogenesis only, exerting its action independently of the VEGF pathway by recruiting endothelial and myeloid precursors [115]. Smaller diameter gliomas unveil lower MMP-2 expression, while in larger diameter malignant gliomas MMP-2 is highly expressed, with corresponding reduction in the expression of tissue inhibitory proteins and tissue inhibitors of metalloproteinases (TIMPs) [116]. Reduced expression of MMP-2, 9, 14, 15, 16, 17, 24, and 25 was revealed in human glioblastoma U373 cells treated with varying concentrations of curcumin in DMSO compared to untreated cells [110]. Cell invasion assays conducted with human glioblastoma cells A1027 and SNB19 showed a significant reduction in cell migration at curcumin concentrations 10 μM, 15 μM, and 20 μM compared to controls [117]. The Wnt pathway is important in adult tissue homeostasis and is able to induce mitogenic stimulation, differentiation, and cell fate specification via a signal cascade that starts on the surface of signal cells that bind to the Frizzled/low density lipoprotein receptor-related protein complex on the target cell, with signal transduction by Disheveled, glycogen synthase kinase-3β, Axin and adenomatous polyposis coli (APC), and following increase in the intranuclear and intracytoplasmic concentration of β-catenin [118]. It has been demonstrated that regulation of the Wnt signaling pathway is important in the regulation of early brain development and is essential for postsynaptic and presynaptic transcriptional regulation, while Wnt dysregulation has been correlated with tumor development, including glioblastoma [119]. Hepatoma-derived growth factor (HDGF) is a growth factor that promotes angiogenesis and is upregulated in gliomas, HDGF eventually forms a complex with β-catenin from the Wnt pathway; this upregulation of Wnt and HDGF develops tumor generation, progression, and metastasis [120]. Human glioblastoma cell lines U251 and LN229—cultured in the presence of curcumin concentrations ranging from 5 to 200 μmol/L—presented reduced invasion, migration, and proliferation distance. Furthermore, curcumin could inhibit HDGF resulting in a reduction in the complex formed between HDGF and β-catenin (which has been demonstrated to promote tumorigenesis) [121].

### 2.2. Flavonoids

Flavonoids are secondary polyphenolic plants metabolites and can be separated into seven different classes: flavones, flavanones, flavonols, flavan-3-oils, isoflavones, anthocyanidins, and chalcones. In flavonoids the supporting structure is polyphenolic, organized by 15 carbon atoms which are connected in a three-ring structure [122]. These compounds have anticancer properties by means of upregulation of apoptosis and inhibition of migration, invasion, and metastasis [123]. Additionally, flavonoids can regulate the glucose metabolism of cancer cells and decrease the Warburg effect. Among the flavonoids with anti-glioblastoma potential are resveratrol, a stilbenoid present in red wine and grapes, and epigallocatechin-3-gallate (EGCG), a catechin present mainly in green tea [124].

#### 2.2.1. Resveratrol

**Figure 3:**
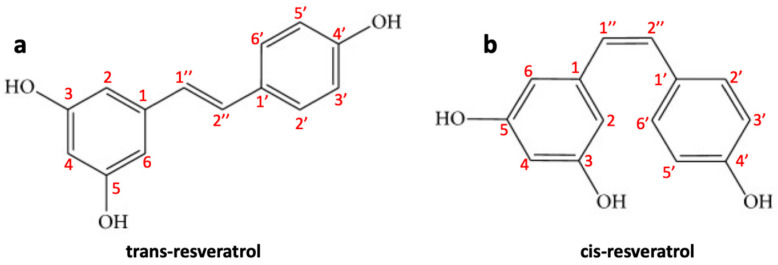
Molecular structure of (**a**) trans-resveratrol and (**b**) cis-resveratrol.

Resveratrol (trans-3,4′,5-trihydroxystilbene) (RES) is a naturally occurring polyphenolic phytoalexin found in peanuts (*Arachis hypogaea*) and grapes (*Vitis vinifera*), mulberries (*Morus* sp.). It is produced by spermatophyte plants triggered by injury, UV radiation, stress, fungal infections (such as *Botrytis cinerea*) and/or other pathogens [125]. In plants, RES biosynthesis occurs through the phenylalanine pathway. Although the presence of a double “bridge” can form both the trans and the cis forms of RES (Figure 3a,b), the trans isomer is sterically more stable compared to cis isomer [126]. RES is a natural pharmaceutical compound with a large range of biological activities such as anti-inflammatory, antifungal, anti-aging, antiviral, and antioxidant effects. In recent years, due to its anti-inflammatory and antioxidant properties, it has attracted great attention for cardiovascular disease treatment [127]. The biological activities of this natural polyphenol are mainly correlated to its unique characteristic structure with multiple hydroxyl phenolic groups as the polyphenolic components can eliminate free radicals with the formation of more stable molecules with low toxicity compared to the original radicals [128]. RES also has chemopreventive and antioncogenic properties that have been shown in different types of cancer. RES has also been reported to prevent tumor initiation, promotion, and progression; for example, its antitumor activity has been investigated in different types of cancer, such as prostate, liver, lung, colorectal, and breast cancer [129]. It has been shown that RES is able to prevent the development of skin cancer in mice during the different stages of carcinogenesis and inhibit breast cancer induced by DMBA (7,12-dimethylbenz [a] anthracene) [130]. Clinical studies assessed on patients with colorectal cancer have revealed that RES can inhibit the proliferation of cancer cells and increasing the levels of caspase-3 in hepatic malignant tissue compared to patients treated with placebo [129]. Furthermore, RES can successfully penetrate the BBB and, therefore, can be employed as an effective therapeutic or protective agent against CNS lesions/disorders and tumors, such as glioblastoma [131]. Some studies have also shown that RES, as a radio/chemo sensitizing agent, can improve the efficacy of radiotherapy/chemotherapy drugs against glioblastoma cells [132]. Below we examine in detail what is the chemopreventive and antitumor role of RES in glioblastoma.

##### Pharmacokinetics RES

RES, after being absorbed, has a half-life of about 8–14 min in the plasma, whereas, for its metabolites it is about 9.2 h. RES binds in the bloodstream to protein transporters, protein, lipoproteins, or serum albumin in the order of high density lipoprotein (HDL) < low density lipoprotein (LDL) < very low density lipoprotein (VLDL) [133]. RES absorption occurs after passive diffusion or by transport with ion channels to penetrate the cytoplasmic membrane, allowing its intracellular biological activity [134]. Upon oral administration, RES is absorbed and piles up in various organs—such as the liver, stomach, or intestine—as sites of its extensive metabolism absorption [135]. RES and its metabolites can be accumulated in target cells or organs of different diseases—including colorectal, breast cancer, or leukemic cell tissue. Moreover, RES and its metabolites pile up in ocular and myocardial tissues after oral administration whereas, no RES accumulation was reported in neuroblastoma tumor tissue in athymic mice [136].

##### Antitumor Properties of RES in Glioma

**Figure 4:**
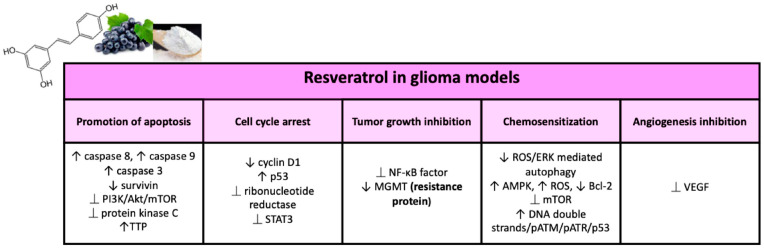
Major effects of resveratrol on glioma models in preclinical studies.

In 1997, Jang et al. for the first time showed the chemopreventive activity of RES in leukemic cells. Since then, RES has revealed diverse anticancer properties in numerous cellular and animal models of glioma, and various mechanisms by which the molecule acts this antitumor activity [137,138] have been reported (Figure 4).

##### Cell Cycle Regulation and Carcinogenesis

In mammals, cell proliferation results in two processes: (1) the cell cycle, including duplication of the genetic heritage and cell division; and (2) cell growth, regulated by multiple growth factors. The four phases of the cell cycle— G1 (Gap 1), S (synthesis), G2 (Gap 2), and M (mitosis)—are principally defined by cyclin-dependent kinases (CDK) which act in a complex with their partners cycling. Cell cycle arrest is an irreversible process and can lead to cell death by apoptosis [139]. RES could delay cell cycle progression and was able to block the proliferation of rat C6 glioma cells at micromolar concentrations, arresting the S-phase cell cycle. The authors also reported that the expression of specific oncogenic microRNAs (miRs)—such as miR-19, miR-21, and miR-30a-5p—in glioma cell inhibition is related to an altered expression of their target genes such as STAT3, p53, EGFR, NF-κB, COX-2, and the PI3K/AKT/mTOR signaling pathway. RES also presents suppression of tumor growth and prolonged survival in rats presenting intracranial C6 glioma [140]. Induction of S-G2/M cell cycle arrest by RES has also been showed in human glioblastoma cell lines, and this has been accomplished by increasing levels of pCdc2 (Y15); cyclin A, B, and E; and by reducing cyclin D1 [141].

Several molecules are responsible of the carcinogenesis and progression of brain tumors, which can be considered therapeutic targets for RES. One of the major targets of the RES antiproliferative activity could be the cytoplasmic tumor suppressor protein p53 since most gliomas have mutations in p53 or alterations in its path. P53 suppresses DNA replication in response to stress and DNA damage in mutant cells, promoting cell cycle arrest in the G1/S phase [26]. RES upregulates p53 protein expression upon promoting its accumulation and phosphorylation. The RES also blocks the progression of the cell cycle by downregulating enzyme cyclin D1 expression, the latter being connected to the transition from phase G1 to phase S of the cell cycle [142]. RES is also capable of inhibiting ribonucleotide reductase, which is required for DNA synthesis in the S phase of the cell cycle, thus inducing cell cycle arrest [143]. Another largely studied target of RES is the transcription factor STAT3, which is correlated in extracellular signals transmission in the nucleus, thus influencing the transcription of several genes [144]. The STAT3 signaling pathway influences recurrence and the malignancy of glioma tumors and is extensively activated by phosphorylation in brain tumors [145]. STAT3 in carcinogenesis upregulates genes that can develop cell cycle progression, tumor survival, angiogenesis, and resistance to cell death. STAT3 upregulation in glioblastoma has been demonstrated in various studies [146]. STAT3 is required for tumor formation and the maintenance of self-renewal of glioblastoma-like stem cells, some of which expresses CD133 as a cancer stem cell marker [147]. RES could act its antitumor activity on glioblastoma CD133^+^ tumor-initiating cells by inhibiting cell viability and growth, blocking self-renewal capacity, inducing apoptosis, and enhancing radiosensitivity in vitro and in vivo through suppression of the STAT3 street [148]. Moreover, in medulloblastoma (commonest type of primary brain tumor in children) RES showed suppression of cell growth through STAT3 downregulation, reducing the incidence of STAT3 nuclear translocation and promoting neuronal differentiation of cell growth medulloblastoma through axon regeneration and SOCS3 accumulation at the synapse-like end of long cellular processes [149].

##### Inhibition of Angiogenesis and Tumor Growth

An aberrant neovascularization and cellular invasion of surrounding tissues are closely connected with the degree of malignancy and invasiveness of glioma tumors. There are several growth factors and cytokines that regulate vascular cell proliferation and the invasion of adjacent tissues [150]. Vascular endothelial growth factor (VEGF) enhances the formation of capillaries by stimulating the proliferation, migration, and invasion of the surrounding tissues—endothelial cells—to form new vessels [151]. Recent studies revealed that RES could inhibit the expression and activity of VEGF in glioma cells, with suppression of angiogenesis [152]. Furthermore, the proteolysis of the extracellular matrix is another crucial event for the infiltrative growth of glioma [153]. It has been demonstrated that RES determines the suppression of the activation of the NF-κB factor, thus hindering the nuclear translocation of its p65 subunit, with a downregulation of the expression of the uPA/uPAR genes and a reduction in the invasiveness of glioma cells [154].

##### Promotion of Apoptosis

Apoptosis, programmed cell death, produces a series of cellular changes, such as loss of integrity of the cytoplasmic membrane, cell shrinkage, and DNA fragmentation that cause cell death [155]. Several extra and intracellular factors can favor apoptosis and the absence of apoptotic responses is essential for tumor development and resistance to therapy. RES has been shown to induce apoptosis via different mechanisms in glioma cells [156]. In 2004, Tseng et al. demonstrated that RES is capable of inducing apoptosis of glioma cells in rat models [152]. Furthermore, RES is able to induce apoptosis by both the caspase-8-dependent and caspase-9-dependent pathways in U251, U87, and C6 glioma cells [157,158]. These are both initiating caspases responsible for activating effector caspase-3 (through hydrolytic cleavage), which disassembles glioma cells into apoptotic bodies [159]. RES can also favor apoptosis by downregulating the expression of an effector caspase inhibitor, surviving [160]. Additionally, recent studies have shown that RES can promote caspase-3 activation in glioma cells by inhibiting the intracellular signaling pathway PI3K/Akt/mTOR [161]. The PI3K/Akt/mTOR pathway is overactive in glioma tumors, resulting in enhanced tumor proliferation and reduced apoptosis, and is frequently implicated in resistance to anticancer therapies [152]. RES can also induce apoptosis through the inhibition of another signaling pathway, the protein kinase C pathway [162]. Protein kinase C has been shown to be overexpressed in glioma cells and related to cell proliferation [163]. Other studies have revealed that RES exerts its antitumor activity also at the level of gene expression post-transcriptional regulation. An RNA-binding protein, tristetraproline (TTP), can bind AU-rich elements in target mRNAs with high affinity, favoring the deadenylation and decay of target transcripts such as antiapoptotic genes, proto-oncogenes, immune regulatory genes, and others [164]. In human U87MG glioma cells, RES presented an increased TTP expression, inducing apoptosis and cell growth inhibition [165].

##### RES and Cellular Senescence

Cell senescence is a non-reversible arrest of the cell cycle, which determines the suppression of the tumor by stopping proliferation. Therapy-induced senescence is thought to be an effective tool in the treatment of cancer, with fewer undesirable effects than treatment that induces death by apoptosis [166]. RES, in a work with human U87 and U118 glioma cells, showed the inhibition of proliferation with induction of cellular senescence in a time- and dose-dependent manner [167]. RES resulted in significant changes in morphology and cell volume; spindle-shaped glioma cells were transformed into flat, hypertrophic cells expressing β-galactosidase (marker of senescence) [168]. In addition, RES resulted in an inhibition of mono-ubiquitination of histone H2B at K120 (uH2B) [166]. In another study, RES-induced senescence in human and rat glioma cells was revealed to be enhanced by inhibition of histone deacetylases [169]. The role of histone deacetylase sirtuin 2 (SIRT2) as a mediator of RES inhibitory activity on glioblastoma stem cell (GSC) proliferation was confirmed by Sayd et al. [170]. RES blockade of GSC cell cycle at concentrations below 150 μM was mediated by SIRT2, while GSC necrosis induced by higher RES concentrations was independent of SIRT2 activity [171].

##### Sensitization to Anticancer Drugs (Such As Temozolomide)

RES can modify multidrug resistance acting as cancer cells sensitizer to standard chemotherapy molecule. The standard drug, used in the treatment of glioblastoma, is temozolomide. Its use, however, is highly ineffective because of various resistance mechanisms and biological barriers [172]. RES is capable of increasing the efficiency of temozolomide through several mechanisms. Temozolomide induces both apoptosis and autophagy in human glioma cells by the activation of the extracellular signal-regulated kinase (ERK) and a burst of reactive oxygen species (ROS). During these events, however, autophagy protects glioma cells from death by apoptosis. It has been shown that RES could increase the therapeutic efficacy of temozolomide through the reduction in ROS/ERK-mediated autophagy following an increase in apoptosis both in vitro and in vivo [173]. In human SHG44 glioblastoma cells, the RES/temozolomide combination has revealed additional antiproliferative effects upon inhibition of mTOR signaling and downregulation of the antiapoptotic Bcl-2 protein and increase in ROS production, and subsequent activation of AMPK. These results were confirmed in a mouse model of orthotopic xenograft showing reduction in tumor volume and reduction in Ki-67 expression (marker of proliferation) [174]. Glioblastoma initiating cells (GICs) showed the properties of stem cells and played an essential role in temozolomide resistance, tumor development, and tumor recurrence [175]. RES resulted in an improvement in the sensitivity of these cells, highly resistant to temozolomide, upon activation of the DNA double strands/pATM/pATR/p53 pathway, resulting in the activation of apoptosis [176]. Additionally, RES promoted GIC differentiation involving p-STAT3 inactivation. It has been shown that a RES dimer, viniferin, is capable of increasing the apoptosis of the glioblastoma cell line induced by cisplatin, another chemotherapeutic agent, in vitro through the activation of caspases 3, 8, and 9 [177]. Therefore, RES in combination with other anticancer agents enhances their effectiveness, either additively or synergistically.

##### Radiosensitization

RES is also able to act as an antitumor radiosensitizing agent in the colon, prostate, skin, breast cancer, leukemia, hepatoma, and other cancers, including brain tumors [42]. RES is also a radiation sensitizer in SU-2 (highly radio-resistant) human glioma stem cells. The radio sensitizing action of RES has been observed in the induction of autophagy, induction of differentiation, inhibition of cell proliferation, promotion of apoptosis, and prevention of early phase DNA repair, both in vitro and in vivo [140].

##### RES and Resistance Proteins

Temozolomide-based chemotherapy after neurosurgery has revealed to be effective, however, due to resistance to temozolomide, not all patients treated benefit from it. The most important feature of resistance to temozolomide is the expression of the O(6)-methylguanine DNA-methyltransferase (MGMT) protein [178]. MGMT repairs DNA damage caused by temozolomide-induced methylation; therefore, it is associated with resistance to temozolomide therapy [179]. Huang et al. demonstrated that RES could decrease the expression of the MGMT protein in glioblastoma cells, with the suppression of the activation of the transcription factor NF-κB which is essential for the activation of the MGMT protein [180]. Consequently, RES enhances the effectiveness of temozolomide therapy, reversing resistance to therapy. Inhibition of the activated Wnt signaling pathway via downregulation of MGMT expression would appear to be another mechanism to inhibit proliferation and induce apoptosis of resistant glioma cells via the RES/TMZ combination [181]. Furthermore, the presence of RES forced various glioblastoma cells—U87-MG, U-138 MG, and U251—treated with temozolomide through mitosis leading to mitotic catastrophe and senescence, with a reduction in the clonogenic capacity of the cells and an increase in the chronic effects of temozolomide [182].

#### 2.2.2. Epigallocatechin Gallate

**Figure 5:**
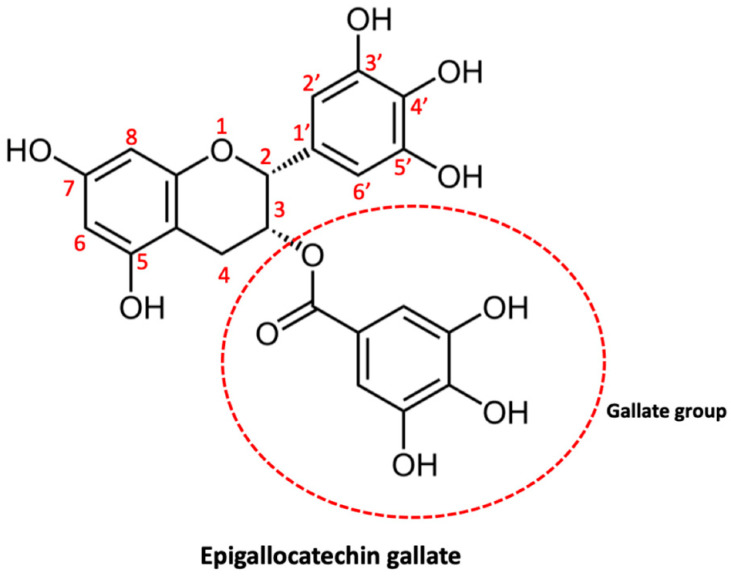
Molecular structure of epigallocatechin gallate.

Epigallocatechin gallate (EGCG) (Figure 5) is a very important polyphenolic component of green tea and is also one of its main catechins. In a variety of diseases, EGCG has been extensively studied for its chemopreventive and chemosensitizing potential [183]. EGCG can bind to GRP78—a crucial pro-survival component of the endoplasmic reticulum (ER) stress response system—and repress its antiapoptotic function, this interaction renders cancer cells more chemo sensitive [184]. There are numerous studies that have shown the ability of EGCG to improve the sensitivity of various types of cancer to various proapoptotic drugs—including taxol, 5-fluorouracil, and gemcitabine—in vitro and to paclitaxel and doxorubicin in vivo [185]. Studies have also demonstrated that EGCG can reverse drug resistance with induction of apoptosis and inhibition of P-gp and ABCG2 expression in drug-resistant cancer cells of the breast, lung, and ovaries [186]. The effects of EGCG on glioma will be examined below.

##### EGCG Pharmacokinetics

Several recent clinical studies have examined the pharmacokinetic profile of oral administration of EGCG in healthy subjects. In 2003 Ullmann et al. studied the tolerability, safety profile, and pharmacokinetic properties of administering a single dose of EGCG ranging between 50 and 1600 mg. For oral doses greater than 1 g EGCG only, maximum plasma EGCG concentrations greater than 1 μM (1600 mg dose) were measured. The peak concentrations were reached between 1.3 h and 2.2 h. For both free EGCG and total EGCG (free EGCG + conjugated EGCG metabolites), plasma kinetics were measured at intervals for a period of 26 h after administration. Under the concentration–time curve from 0 h to infinity (AUC_(0–∞)_) the mean total EGCG area ranged between 442 ngh/mL and 10,368 ngh/mL, while the mean terminal elimination half-life t_1/2z_ ranged between 1.9 h and 4.6 h. Generally, doses of purified EGCG up to 1600 mg were well tolerated [187]. While, in 2003, Chow et al. examined the plasma kinetics and safety of multiple dose administration of purified EGCG. This study evaluated once-daily and twice-daily EGCG dosing regimens over a four-week period. As was observed in the previous study, also in this case the intake of EGCG at doses of 400 mg and 800 mg resulted in peak serum concentrations of both free and total EGCG in the high nanomolar range. While an increase in the bioavailability of EGCG was registered upon chronic administration of 800 mg. Finally, the daily administration of EGCG resulted in only mild gastrointestinal side effects [188]. In another work, Lei-Chwen et al. evaluated the pharmacokinetics of EGCG in conscious, free-moving rats. The limit of quantification (LLOQ) of EGCG in rat plasma was determined in 5 ng/mL. While the brain distribution result clearly showed that EGCG could potentially penetrate at a slower rate through the BBB. Specifically, the distribution for the different brain regions was as follows: cerebellum 7.13 ng/g; cortex 6.23 ng/g; striated 4.72 ng/g; hippocampus 4.18 ng/g; brain stem 3.76 ng/g; rest of brain 1.31 ng/g, 15 min after IV administration of 50 mg/kg EGCG. Thus, the concentrations of EGCG in the different brain regions were around the LLOQ [189]. Furthermore, although the lipophilicity of the EGCG chemical groups suggests passive permeability through the BBB, Nakagawa and Miyazawa measured an EGCG concentration of 0.5 nmol/g in rat brain tissue 60 min after oral administration of 500 mg/kg. This reduced cerebral localization can be attributed to the bipolar functional group of the EGCG, which may have difficulty crossing the BBB [190].

##### Antitumor Properties of EGCG in Glioma

**Figure 6:**
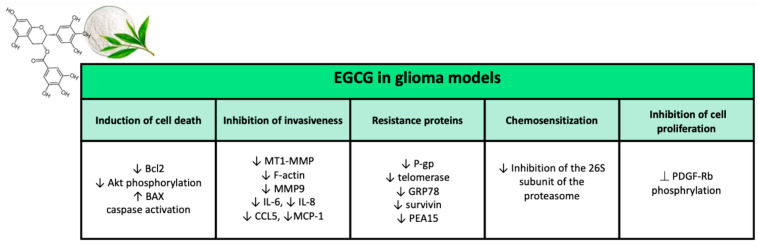
Major effects of epigallocatechin gallate on glioma models in preclinical studies.

EGCG possesses a wide range of biological activities that may have significant effects on cancer cells. Reports from several clinical trials on the chemopreventive potential of EGCG in cancer started to be published [191]. This review focuses mainly on the effects of EGCG in glioma, which are summarized in Figure 6.

##### Inhibition of Cell Proliferation

EGCG can block the proliferation of several types of human cancer cells in vitro, including glioma cells. Cell proliferation, apoptosis, and invasiveness of glioma cells are affected by EGCG. On the part of EGCG, one of the mechanisms of inhibition of cell proliferation is affecting PDGF pathway [192]. Through the autophosphorylation of tyrosine residues, upon binding to the PDGF-BB ligand, the b receptor of PDGF (PDGF-Rb) transmits mitogenic signals. The role of PDGF-BB and its receptor in cell proliferation has been examined in several types of cancers, including glioma, colon cancer, sarcoma, melanoma, and breast cancer [193]. In detail autocrine activation of PDGF-Rb has been revealed to be involved in the development and progression of glioma. Treatment of glioblastoma A172 cells with EGCG (50 µM) resulted in a significant suppressing of PDGF-BB-induced PDGF-BB tyrosine residue phosphorylation. Blocking of PDGF-Rb corresponded to suppression of spheroid formation of A172 cells [194]. It has also been reported that EGCG is able to suppress PDGF-induced proliferation in other types of cancer through the inhibition of PDGF-Rb phosphorylation. Furthermore, EGCG with its galloyl group has been shown to interact directly with PDFG-BB, preventing PDFG-BB from binding to PDGF-Rb [195]. Consequently, EGCG is likely to inhibit PDGF-Rb phosphorylation in glioma cells upon blocking the binding of PDFG-BB to its receptor. The cytotoxic activity of a drug is generally evaluated through its effects on cell viability. The activity of metabolically active proteins is evaluated by viability assays. Tetrazolium compounds—such as MTS, MTT, and WST-8—are often used in such assays, as they are reduced by viable cells to their purple formazans. In addition, cell viability can also be assessed with ATP quantification assays [196]. Various works have shown that EGCG can inhibit the viability of several human glioma cell lines (U87, U87 GSLC, U251, MO59J, U373, LN18, 1321N1, SW1783) and the rat glioma cell line C6. To evaluate the long-term effects of EGCG treatment, colony formation assays were examined. In the work of Golden et al., EGCG at concentrations of 10–20µM did not result in inhibition of U251 and LN229 glioma cell colony formation [197], while dose-dependent inhibition by EGCG, of colony formation, was confirmed by Chen et al. (concentrations between 0 µM and 100 µM of EGCG applied to U251 and U87 cells) [198] and by Zhang et al. (concentrations between 0 and 200 mM of EGCG applied to U87 and U87 GSLC) [199]. U87 GSLCs express stem cell markers, such as CD133 and ALDH1, which constitute the tumorigenic cell population in glioblastoma with unlimited proliferation potential to support maintenance and tumor development [200]. All these results further confirmed the cell proliferation inhibitory activity of EGCG on glioma cells.

##### Induction of Cell Death

A drug can exert its cytotoxic activity through the induction of cell death. Various tests can be used to detect dead cells, which are based, for example, on deoxynucleotidyl transferase dUTP nick-end labeling (TUNEL), trypan blue, propidium iodide (PI), annexin-V, and Wright’s stain [201]. Several works have shown the tumoricidal activity of EGCG on human glioma cell lines (A172, U87, U251, U373, MO59J, T98G) and on the rat glioma cell line C6, while tumor cells of the pituitary gland of rat (MtT/E) and normal human astrocytes were resistant to the effects of EGCG [202,203]. Another system for evaluating cell death is the measurement of the potential of the mitochondrial membrane, the activity of anti-apoptotic proteins, and the activity of proteins associated with apoptosis. In this respect, upon treatment with EGCG, an interrupted mitochondrial potential, free calcium, split BID, and split PARP, reduced levels of Bcl-2 and phosphorylated Akt and increased levels of BAX, activated caspases were measured [199,204]. There is controversy in the literature about the pro-apoptotic potential of EGCG. While Chen et al., using the PI and TUNEL assays, did not report an increase in death on U87 cells after 48 h of treatment with 20 mM EGCG [198], McLaughlin et al. instead observed EGCG cell death inducing effects on U76 cells for lower EGCG concentrations and shorter incubation periods (6 h) [205]. It follows that it is important that these studies should be repeated; however, given the large number of studies demonstrating the cytotoxic effect of EGCG, it seems appropriate to state that EGCG is capable of inducing cell death in glioma cells in vitro.

##### Inhibition of Invasiveness

Glioma tumors are characterized by a high invasiveness, limiting the effectiveness of surgery and radiotherapy. Hence, suppression of the invasiveness of glioma cells is of the utmost importance for the outcome of patients with glioma, as it permits more radical local therapy [206]. To examine cell invasiveness, one method is the Matrigel test, several studies have reported an inhibitory effect of EGCG on the invasiveness of various glioma cell lines (U87, U87 GSLC, and U251). EGCG can block the invasiveness of glioma cells through the inhibition of MT1-MMP [202]. EGCG reduces the expression of MT1-MMP by gene silencing. Upon activation of pro-MMP2 in MMP2, MT1-MMP promotes the invasion of glioma cells. A significant dose-dependent inhibition by EGCG on MMP2 activity in U87 was found thanks to gelatin zymography. Even through exposure of U87 cells to concanavalin A (activator of pro-MMP2 and strong inducer of MT1-MMP expression), EGCG could still deactivate pro-MMP2 in MMP2 via MT1-MMP. Since MMP2 is one of the main proteases involved in the invasive behavior of cells, the inhibition of the activation of MMP2 through MT1-MMP by EGCG determines a reduction in the invasiveness of glioma cells [207,208,209]. Furthermore, MT1-MMP can regulate the invasiveness of glioma cells via CD44 expression and restructuring of the actin cytoskeleton. CD44 is a glycosylated transmembrane receptor for various components of the extracellular matrix, including osteopontin and hyaluronic acid (HA). Tumor migration was shown to be promoted by both CD44 and its HA ligand [210]. By binding to CD44, HA promotes the cleavage of CD44 from the cell surface, an event that is fundamental for cell detachment and invasive cell behavior [211]. MT1-MMP, with its cytoplasmic domain, regulates the MAPK signaling pathway, resulting in deep restructuring of the cytoskeleton and increased expression of RhoA. The increase in RhoA expression involves the activation of ROCK which in turn phosphorylates the cytoplasmic domain of CD44, facilitating CD44/HA binding. Therefore, through the blocking of the expression and activity of MT1-MMP, the EGCG determines a reduction in the cleavage of CD44 induced by HA [212]. Furthermore, it has been reported that the suppression of the invasiveness of U87 cells by EGCG occurs through the reduction in F-actin levels, following a disruption of the architecture of the cytoskeleton. Therefore, MT1-MMP—through the MAPK pathway—is capable of influencing the architecture of the actin cytoskeleton thus facilitating cell migration, so that the inhibition of MT1-MMP by EGCG can lead to an interrupted network of actin [204]. In summary, EGCG limits the invasiveness of glioma cells by blocking the cleavage of CD44 and disturbing the restructuring of the actin cytoskeleton through inhibition of MT1-MMP [212]. Furthermore, EGCG inhibits MMP9 activity in U87 and U251 glioma cells. It has been reported that the increase in MMP9 expression is associated with an increase in the invasiveness of U251 cells [213]. Agarwal et al. showed a significant (two-fold) reduction in levels of interleukin 6 (IL-6), IL-8, chemokine ligand 5 (CCL5 or RANTES), and monocyte chemotactic protein 1 (MCP-1) upon treatment with 100 mM EGCG of U87 cells [214]. Such chemokines and cytokines are able of inducing invasiveness of glioma cells. IL-6 is a pro-inflammatory cytokine that plays a role in the progression of higher-grade glioma astrocytoma with the induction of VEGF expression [215]. In addition, IL-6 can lead to increased invasiveness of U87 cells through the upregulation of MMP-2 [216].

##### Chemosensitization

TMZ is a chemotherapy alkylating agent employed in the treatment of newly diagnosed glioblastoma; however, as discussed, its effect is reduced to only a few months of prolonged survival, confirming the urgent need to identify treatment options that are more efficient [217]. In addition to the synergy with TMZ, EGCG also improved the effect of cisplatin, tamoxifen, and carmustine (BCNU) in various glioma cell lines [198]. Like TMZ, carmustine is also an alkylating agent, while cisplatin causes damage to cellular DNA and tamoxifen is capable of inhibiting the cellular proliferation of cancer cells by blocking estrogen receptors [218,219]. The same effects were also revealed in U87 cells grown as neurospheres. Another chemotherapeutic agent is the ligand that induces apoptosis related to TNF (TRAIL), it represents a considerably safe and promising chemotherapy agent for the specific triggering of apoptosis in cancer cells through death receptors. Cancer cells, however, often develop resistance against TRAIL over time. A study by Siegelin et al. reported that EGCG could enhance TRAIL-mediated apoptosis in three different human glioblastoma cell lines, regardless of TP53 status [220]. While, on the other hand, another study showed that EGCG results in a reduction in the therapeutic effects of bortezomib (BZM) on glioma cells. BZM is a selective inhibitor of the 26S proteasome, responsible for the degradation of ubiquitinated proteins. Inhibition of the 26S subunit of the proteasome has a huge number of consequences, including excessive accumulation of misfolded proteins, which ultimately generates endoplasmic reticulum (ER) stress and cell death. Golden et al. examined the effect of EGCG on LN229 and U251 cells in combination with BZM using two assays: the MTT assay to study the immediate effect on cell viability and the colony formation assay to assess the long-term effect on survival [197]. In both experimental settings, EGCG resulted in an effective blocking of the inhibition of the proteasome by the BZM, thus reducing the effectiveness of the BZM in determining the death of glioma cells. The antagonism of EGCG and BZM has also been demonstrated in other cell lines in independent studies [221]. It is hypothesized that EGCG with the boronic acid fraction of BZM forms a stable cyclic boronate adduct, thus not permitting BZM to act as a proteasome inhibitor [197]. Through a similar mechanism, EGCG inhibits the effects of an ER stress-inducing agent, fenretinide [221]. Therefore, in summary, EGCG can improve the effects of various chemotherapeutic agents; however, the potential antagonistic interactions between EGCG and other drugs must also be considered.

##### Radiosensitization

McLaughlin et al. exploited whether EGCG treatment was able to potentiate the anti-glioma effects of radiotherapy employing U87 glioma cells [205]. At first, the radio-resistant properties of U87 cells have been demonstrated by employing high doses of infrared radiation (IR) (10–30 Gy) and comparing with other cell lines such as U118, U138, and DAOY. For U87 cells, a 40% decrease in the proliferation rate was investigated upon exposure to 10 Gy IR and a 50% decrease after exposure to 30 Gy IR. While the combined treatment of EGCG and 10 Gy IR gave rise to a 60% reduction in the proliferation rate of U87 cells, whereas the cells treated with EGCG only or IR only showed a reduction in the proliferation rate of 35% and 40% respectively. Thus, EGCG could potentially be used to increase IR anti-glioma. However, it has been revealed that EGCG is surprisingly able to protect DU145 cells from IR [222]. Thereby, it is extremely important to identify the underlying mechanisms explaining different effects of EGCG on various cell lines to avoid radiation protection of glioma by EGCG rather than radio sensitization.

##### EGCG and Resistance Proteins

A major problem in glioma is chemoresistance to treatment. P-glycoprotein (P-gp) is a multidrug resistance transmembrane protein that pumps various toxic compounds out of cells, as well anticancer drugs [223]. In one paper, Zhang et al. reported that EGCG is able to inhibit the activity of P-gp. Both P-gp protein levels and mRNA levels were reduced after treatment with EGCG in a dose-dependent manner. Therefore, it may follow a further explanation of the synergy between chemotherapy (e.g., with temozolomide and BCNU) and EGCG [198]. In cancer biology, a central dogma is that high levels of telomerase ensure the maintenance of telomere length. In addition, telomerase levels in glioma are often associated with tumor progression and grade [224]. With their work, Shervington et al. demonstrated that the addition of EGCG was capable to cause a significant reduction in telomerase mRNA expression in glioma cells. Blocking of telomerase can reduce telomeres to a critical length, resulting in genome instability and lately activation of the apoptotic pathway. This phenomenon may explain EGCG effects in sensitizing therapy in cancer, including glioma [225,226]. GRP78 is a chaperone protein with various anti-apoptotic functions, such as binding and prevention of caspase-7 activation. Elevated levels of GRP78 expression are correlated with poor outcomes in patients with glioblastoma [227]. Inhibition of GRP78 can be considered an attractive means for inducing chemo sensitization [228]. EGCG was able to interact with GRP78 in the ATP binding domain, reducing the activity of the protein [229]. McLaughlin et al. have shown that EGCG could determine an improvement in the efficacy of anti-glioma therapies, in particular IR, through the downregulation of the expression of the pro-survival survivin protein [205]. Survivin would result in increased radio resistance in human glioblastoma cells. IR induces the expression of survivin with caspase-mediated inhibition of apoptosis by binding and suppressing smac-Diablo or caspase-9 [230]. In other studies, the sensitizing effect of EGCG to TRAIL in glioma cells, mediating suppression of the expression of survivin, was confirmed. Siegelin et al. showed that 24-h treatment of U87 and A172 cells with 20 mM EGCG resulted in a significative reduction in both survivin and PEA15 levels, through sensitization of TRAIL-resistant glioma cells to TRAIL treatment. PEA15 is a caspase-8 inhibitor and is upregulated in various glioma cell lines and correlates with the resistance of human glioma cells to anticancer drugs, such as TRAIL. It has been revealed that the downregulation of PEA15 and survivin by EGCG is obtained through a reduction in Akt phosphorylation in the U87 cell line [220]. Furthermore, a downregulation of telomerase expression was also observed by suppressing Akt phosphorylation. Hence, it seems that EGCG can sensitize glioma cells to anticancer therapies by reducing Akt phosphorylation in glioma cells.

Table 1 summarizes some of the main anti-glioma effects of the natural compounds examined, observed in preclinical studies.

## 3. Challenges and Considerations in the Use of Natural Substances in the Treatment of Glioma

Bioavailability, metabolism, and permeability of BBB are crucial factors in the clinical feasibility of natural compounds in the treatment of glioma. These properties vary largely between substances and in many cases must be exceeded to achieve sufficient clinical and in vivo concentrations [231]. Oral bioavailability is a measure of the ability of a compound to reach the systemic circulation upon ingestion and is deficient in various natural compounds [232]. Curcumin, for example, has a limited oral bioavailability since its poor absorption and rapid first-pass metabolism. Therefore, although increasing evidence shows that curcumin is capable of crossing the BBB, its limited solubility in water, hydrophobic nature, and physico-chemical stability reduce its bioavailability [233]. Thus RES, despite its promising potential as a therapeutic agent, faces challenges in its administration due to various limitations. RES exhibits low bioavailability due to its rapid metabolism and clearance, short biological half-life, poor solubility in water, chemical instability, and high photosensitivity. RES once reached the bloodstream is quickly absorbed: it is metabolized in the liver and very fast eliminated [234]. Furthermore, the healthful effects of EGCG are hindered by its degradation in biological fluids, or during the processing and storage of tea. This degradation happens in two main reactions: auto-oxidation and epimerization. Additionally, it has been reported that the oral bioavailability of EGCG is low, both in animal models and in humans, due to its high molecular weight and metabolic alterations [235,236]. Therefore, the low bioavailability hinders the accumulation of these compounds in target tissues at the concentrations necessary for therapeutic success. Consequently, because the success of a therapy for CNS disorders largely depends on the bioavailability of the therapeutic agent, new therapeutic approaches are necessary. Currently, considerable efforts are being devoted to the development of new delivery systems to overcome these limits, and at the same time to enhance the therapeutic efficacy of natural compounds. There are several types of materials that have been used for the encapsulation of natural compounds and the increasing of their activity [237]. Some of the proposed systems have advantageous characteristics, such as bioavailability, improved stability, and biocompatibility. For example, silica nanoparticles (NPs) significantly enhanced the biological activity and carrying capacity of the RES [238]. There are some publications where authors have examined the effectiveness of RES delivery systems in the treatment of glioblastoma. For example, Figueiro et al. investigated the antitumor effect of RES-loaded lipid core nano capsules against C6 glioma cells in both in vitro and in vivo models. In vitro, RES-loaded NPs limited the viability of cancer cells to a greater extent respect to free RES upon induction of cell death by apoptosis. In vivo, treatment with the nano-complex produced a significant reduction in tumor size respect to free RES [239]. For example, when loaded into casein NPs, the oral availability of RES increased by up to 10 times [240]. Curcumin nano formulated with poly-lactic-co-glycolic acid (PLGA) in a mouse model showed three- to four-times greater longevity and concentration in CNS tissue at similar oral doses compared to standard curcumin preparations [241]. Liposomes have been used as drug delivery systems for decades. Liposomal curcumin enhanced the bioavailability of curcumin [242]. Curcumin micelles are another form of higher bioavailability of curcumin, approximately 19-times greater than standard preparation curcumin [243]. Many studies have showed that the encapsulation of EGCG within a polymer matrix, such as chitosan-based nanocarriers, enhances its pharmacokinetics and pharmacodynamics [244]. Thus, the potential of EGCG in clinical applications is improved by these technologies.

Furthermore, in the design of nanocarriers, biological barriers must be taken into account. Among the existing biological barriers, the BBB is one of the major obstacles for delivery into the CNS. The BBB is made up of a monolayer of specialized capillary endothelial cells that hinders the passage of more than 98% of small drugs and 100% of large ones (>500 Da), protecting the cerebral microenvironment from toxic agents and substances present in the bloodstream [245]. Hence, BBB limits the development of new therapies for CNS disorders. The ability of NPs to overcome the BBB depends on their physicochemical properties and on their ability to interact with the receptors expressed at the level of the BBB. Several researchers have been able to develop nano systems that are efficient in encapsulating natural compounds and delivering them into the brain [246]. Modifying the surface of the NPs can improve their transport through the BBB. The modification strategies can employ functionalization of the nanosystem, with different fractions improving the recognition of NPs by the receptors present on the BBB and permitting targeting of the brain tissue, with the active transport of the nanocarrier through the BBB [247]. Furthermore, the use of positively charged targeted ligands promotes their interaction with the luminal surface of the BBB cells by electrostatic forces. The ligand must be chosen carefully as its receptor must be overexpressed on the BBB and should also preferably be specific for brain tissue to limit the harmful effects on healthy tissues and enhance the accumulation of the therapeutic agent in the brain parenchyma [248]. Thereby, also because in general natural substances are capable to exert an effective therapeutic action only if administered at high doses (primarily due to their hydrophobic nature), it would permit a reduction to a minimum of the quantity and frequency of administration of the therapeutic agent, reaching at the same time a similar pharmacological profile and a reduction in unwanted side effects [249]. For example, the apposition of a transport molecule, such as p-aminophenyl-α-D-mannopyranoside (MAN), to the liposomal curcumin improved its transport through the BBB. Additionally, MAN-modified liposomes appear to preferentially target the brain stem, cortex, cerebellum, hippocampus, and pontine nuclei [242]. However, further studies are necessary to validate the efficacy of nanotechnology-based natural compound delivery systems in the treatment of glioma.

## 4. Concluding Remarks and Perspectives

Natural substances could represent an integral approach in the treatment of chronic diseases such as glioma. Over the past 20 years, extensive research has demonstrated the beneficial effects of phytochemicals in a wide range of human diseases, including brain tumors, and in particular gliobastoma. As reported, members of the curcuminoid families (such as curcumin) and flavonoids (such as RES and EGCG) exert multiple chemotherapeutic effects on glioma both in vitro and in vivo. These compounds exert these effects through various mechanisms which include the regulation of cell proliferation and cell cycle progression, induction of autophagy, apoptosis pathways, inhibition of angiogenesis, and metastasis. Furthermore, the synergistic effects of natural compounds in combination with chemotherapy (chemosensitization) and radiotherapy (radiosensitization) have been confirmed. However, although the results of in vitro and animal model studies are promising, to date they are still not confirmed by clinical studies. In this regard, currently for the substances examined, only one clinical study is available (NCT01712542) which examined the action of curcumin on 13 patients with newly diagnosed pre-operative glioblastoma. This study found the highest serum and tumor concentrations of curcumin using a micellar formulation of curcumin (NovaSol). It has been shown that the curcumin concentration measured in the tumor (mean, 56 pg/mg) may be not sufficient to determine a short-term anticancer effect. While it can be useful for controlling long-term tumor growth. Additionally, the side effects of taking curcumin were less severe than those seen with current medications. As a result, oral administration of micellar curcumin is relatively safer and more tolerated. However, the present clinical study involved a small number of patients and employed a limited dose of curcumin. Therefore, other clinical studies should be carried out with the aim of strengthening their statistical validity [250].

In addition, it is important to underline that the medical clinical feasibility of natural compounds remains severely hindered by their pharmacodynamic and pharmacokinetic properties. Effective therapies in the treatment of glioma require an appreciable oral bioavailability, permeability of the BBB, and selectivity in targeting tumor tissue; the substances tested are insoluble in water and gastrointestinal fluids and have a low oral absorption, resulting in reduced bioavailability, which hinder their clinical application. Therefore, before the introduction of natural substances in the clinical management of brain tumors, several important questions must be answered. Given the number and complexity of cancer-related signaling pathways influenced by natural compounds, further investigations are needed as of yet to overcome pharmacokinetic limitations such as poor bioavailability in humans, to accurately describe their anticancer mechanisms of action, and to define their effectiveness in various brain tumors. With the aim of increasing the bioavailability of natural compounds in organisms and their potential as adjuvant drugs in clinical oncology, future research should focus on the design and development of delivery systems for natural substances, formulations, dosing protocols, possible interactions with other anticancer drugs, on modulations of the metabolism of tumor cells, increase in bioavailability in organisms and stability which should lead to improved antitumor activity. In this context, the use of NPs represents a beneficial approach for the transport of natural compounds to their site of action. Most nano formulations have a controlled release profile, high stability, and load carrying capacity. Different formulations are proposed to enhance their pharmacological properties but are not yet clinically validated. In conclusion, innovative biochemical formulations could enhance their physiological properties of natural compounds and clinical studies could confirm their anticancer efficacy.

## Figures and Tables

**Table 1:** Main anti-glioma effects observed in preclinical studies for curcumin, RES, and EGCG.

Groups of Natural Compounds	Natural Compound	Cell Lines/Model	Effect	Anti-Cancer Mechanism Proposed	Reference
	Curcumin	U87MG, U373, T67, T98G, and C6 cell lines	inhibition of cell survival	suppression of NF-κB and inhibition of the AP-1 signaling pathway	[66,67,68]
	Curcumin	Tu-2449, Tu-9648, and Tu-251 glioma cell lines	inhibition of invasiveness	inhibition of the JAK/STAT3 pathway	[71]
	Curcumin	human primary (A-172, MZ-18) and recurrent glioblastoma lines (MZ-54, MZ-256, MZ-304)	inhibition of cell proliferation	decrease in intracellular STAT3 levels	[72]
	Curcumin	U251 glioma cells	induction of cell cycle arrest in G2/M	increased p53 protein levels	[77]
	Curcumin	U87MG cells	promoting cell cycle arrest	downregulation of cyclin D1 with upregulation of p21	[79]
Curcuminoids	Curcumin	U251	induction of cell cycle arrest in G2/M	increased expression of the DAPK1 protein	[82]
	Curcumin	U87-MG and U373-MG cell lines	promoting of cell cycle arrest in G2/M	promotion of mTOR dependent ATG	[88]
	Curcumin	Rat F98 and mouse GL261	induction of ATG	activation of the mTOR-dependent ATG pathway	[89]
	Curcumin	C6 and U251MG cell lines	induction of arrest in G2/M and autophagy	inhibition of constitutive activation of the PI3K/Akt/mTOR pathway	[91]
	Curcumin	GSCs	suppression of stem-like features with stimulation of ATG-dependent differentiation of GSCs	induction of mTOR-dependent ATG	[99]
	Curcumin	U51, U87, and U235 cell lines	induction of apoptosis	STAT3 inhibition	[109]
	Curcumin	U87MG and T98G cell lines	improved cytotoxic and apoptotic promoting action of TMZ and etoposide	downregulation of mRNA encoding p53 and upregulation of BAX-Bcl2	[112]
	Curcumin	U373 cell line	inhibition of invasiveness	reduction in the expression of MMP-2, 9, 14, 15, 16, 17, 24, and 25	[110]
	Curcumin	U251 and LN229 cell lines	reduced distance of invasion, migration and proliferation	inhibition of HDGF	[121]
	RES	U87 cell line	induction of S-G2/M cell cycle arrest	reduction in cyclin D1	[141]
	RES	Daoy, UW228-2, and UW228-3 medulloblastoma cell lines	cell growth suppression	STAT3 downregulation	[149]
	RES	RT-2 glioma cell line	suppression of angiogenesis	inhibition of VEGF expression	[152]
	RES	U373MG human glioma cell	reduction in cellular invasiveness	suppression of activation of the NF-κB factor	[154]
	RES	U251, U87, and C6 cell lines	induction of apoptosis	induction of caspase-3 activation	[157,158]
	RES	U87MG cell line	induction of apoptosis and inhibition of cell growth	increased expression of TTP	[165]
	RES	SHG44 cell line	enhanced the antiproliferative effects of TMZ	activation of AMPK, inhibition of mTOR signaling, and downregulation of the antiapoptotic protein Bcl-2	[174]
	RES	T98G cell line	improved efficacy of TMZ therapy	reduced expression of the MGMT protein with suppression of the activation of the transcription factor NF-kB	[180]
Flavonoids	EGCG	Spheroids of A172 cells	suppression of spheroid formation	inhibition of phosphorylation of PDGF-BB tyrosine residues	[194]
	EGCG	U87MG cell line	induction of apoptosis	reduced levels of Bcl-2 and phosphorylated Akt and increased levels of BAX, activated caspases and increased ROS	[204]
	EGCG	U87 cell line	reduction in the invasiveness of glioma cells	inhibition of MMP2	[208]
	EGCG	U87 cell line	inhibition of the invasiveness	reduction in levels of IL-6, IL-8, CCL5, and MCP-1	[214]
	EGCG	1321N1 and U87-MG cell lines	improved cytotoxic effect of cisplatin and tamoxifen	suppression of telomerase	[226]
	EGCG	U87 and A172 cell lines	promotion of TRAIL-mediated apoptosis	reduction PEA15 levels	[220]

## Abbreviations

RES	Resveratrol
EGCG	Epigallocatechin-3- gallate
CUR	Curcumin
BBB	Blood–brain barrier
WHO	World Health Organization
CNS	Central nervous system
TMZ	Temozolomide
GCS	Glioblastoma cancer stem cells
ATG	Autophagy
NPs	Nanoparticles
